# A three-armed cryptand with triazine and pyridine units: synthesis, structure and complexation with polycyclic aromatic compounds

**DOI:** 10.3762/bjoc.14.115

**Published:** 2018-06-06

**Authors:** Claudia Lar, Adrian Woiczechowski-Pop, Attila Bende, Ioana Georgeta Grosu, Natalia Miklášová, Elena Bogdan, Niculina Daniela Hădade, Anamaria Terec, Ion Grosu

**Affiliations:** 1 Babes-Bolyai University, Faculty of Chemistry and Chemical Engineering, Department of Chemistry and SOOMCC, Cluj-Napoca, 11 Arany Janos str., 400028, Cluj-Napoca, Romania; 2 National Institute for Research and Development of Isotopic and Molecular Technologies, 67-103 Donath str., RO-400293, Cluj-Napoca, Romania; 3 Department of Chemical Theory of Drugs, Faculty of Pharmacy, Comenius University in Bratislava, Kalinčiakova 8, 83104, Bratislava, Slovakia

**Keywords:** aromatic guests, aromatic nucleophilic substitution, cryptand, NMR titration, 1,3,5-triazine

## Abstract

The aromatic nucleophilic substitution reaction based synthesis of a three-armed cryptand displaying 2,4,6-triphenyl-1,3,5-triazine units as caps and pyridine rings in the bridges, along with NMR, MS and molecular modelling-based structural analysis of this compound are reported. Appropriate NMR and molecular modelling investigations proved the formation of 1:1 host–guest assemblies between the investigated cryptand and some polynuclear aromatic hydrocarbons or their derivatives.

## Introduction

Cryptands with *C*_3_-symmetric aromatic reference groups are exciting targets, on the one hand due to the challenges encountered in their synthesis and on the other due to their ability (macrobicyclic effect [1]) to form supramolecular assemblies with cations, anions or neutral guests, e.g., aromatic molecules [2–6]. The supramolecular architectures involving cryptands (including metallomacrocycles) and aromatic guests are targeted for investigations of aromatic–aromatic contacts [7–8] and for various applications in molecular electronics [9–10]. The recently reported cage-box [11] is able to complex a plethora of aromatic compounds (e.g., anthracene, pyrene, perylene), while in the cases of several metallo-based cryptands the formation of layered host–guest supramolecular structures (with many guests in the cavity of the host) were reported [12–15].

**Figure 1:**
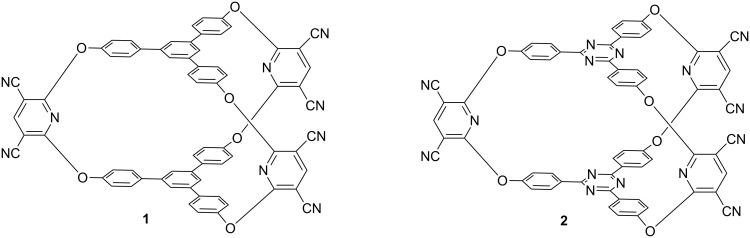
Cryptands with 1,3,5-triphenylbenzene (**1**) and 2,4,6-triphenyl-1,3,5-triazine (**2**) aromatic reference groups.

The synthesis of cryptands with *C*_3_ symmetry by peculiar reactions (acetylenic coupling [16–18], CuAAC [19–22], double or triple bond metathesis [23–25], aromatic nucleophilic substitutions [26–33], or via the amplification of a cryptand belonging to DCC libraries [2,25,34–35]) allowed accessing of more sophisticated architectures. In a previous work [32] we reported the formation of a host–guest complex between a cryptand having pyridine units in the bridges and 1,3,5-triphenylbenzene caps (**1**, Figure 1). Surprisingly, no complexation ability of cryptand **1** towards aromatic guests (anthracene, pyrene) in solution was observed and the formation of a cryptand–pyrene complex was revealed by cyclic voltammetry only after the adsorption of the cryptand on a graphite surface (electrode). In a more recent paper [33], authors who reobtained our cryptand **1** [32] revealed its selective absorption ability for N_2_ and CO_2_ in the solid state. The low complexation ability of cryptand **1** in solution for aromatic guests is due to the unfavorable conformation of the 1,3,5-triphenylbenzene central units in which the peripheral aromatic rings are twisted with respect to the central benzene ring. In such twisted conformations there is a remarkable steric hindrance between the *ortho* hydrogen atoms of the central benzene ring and the *ortho*' hydrogen atoms of the peripheral aromatic units. These conformations are not favorable for a complexation of aromatic guests and the host–guest interactions must be able to equilibrate the previously described *ortho*–*ortho*' hindrance and to bring the aromatic rings of the caps to coplanar conformations. In this work, we decided to investigate a new cryptand in which this steric hindrance is removed. Thus, we changed the design of the cryptand by replacing the central benzene ring with a triazine unit (in which the H atoms of the *ortho* positions are replaced by lone electron pairs belonging to the N atoms). The resulting less sterically hindered cryptand **2** (Figure 1) was then tested for its ability to form host–guest complexes with various aromatic guests.

## Results and Discussion

**Scheme 1:**
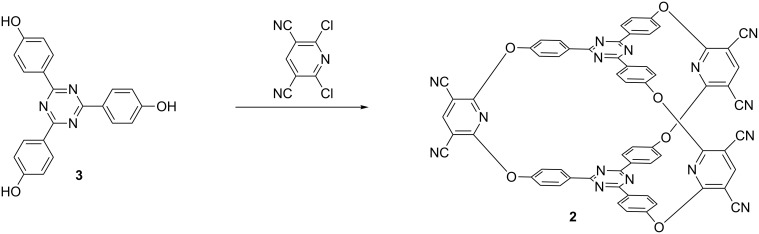
Synthesis of cryptand **2**.

**Figure 2:**
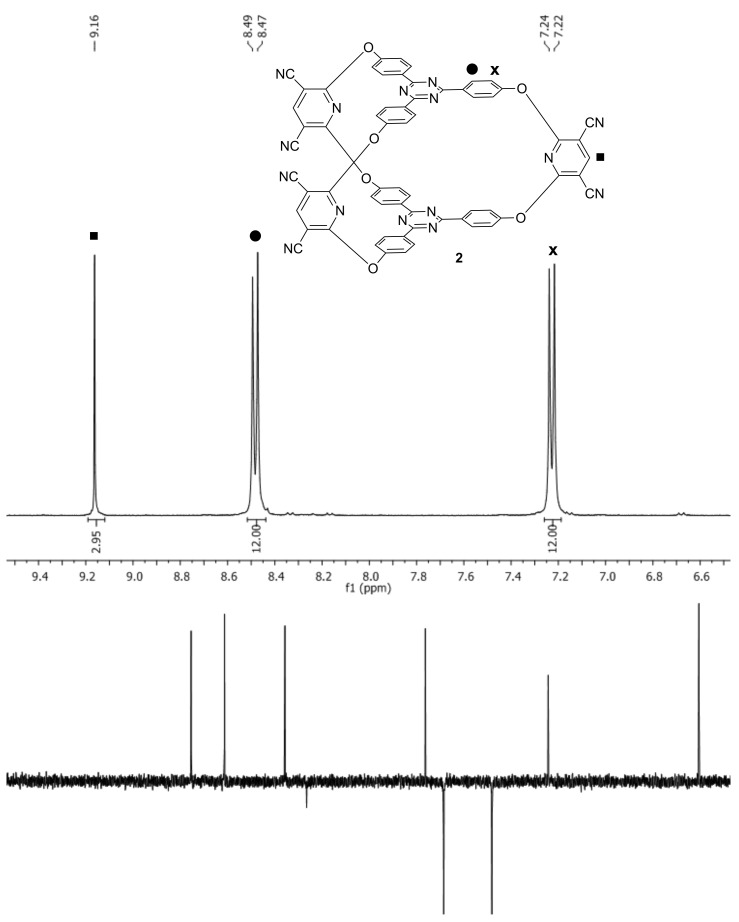
NMR spectra of cryptand **2**: top, ^1^H NMR; bottom, ^13^C NMR.

Cryptand **2** is accessible via nucleophilic aromatic substitution in good yields (42%, Scheme 1) by treating triphenol **3** [36] with the reactive and commercially available 2,6-dichloro-3,5-dicyanopyridine following a procedure previously elaborated in our group [32]. The ^1^H and ^13^C NMR spectra of **2** are quite simple and exhibit a reduced number of signals corresponding to the high symmetry of the target cryptand (Figure 2). In addition MS measurements confirmed the molecular formula of **2**.

Next the complexation ability of cryptand **2** towards pyrene, anthracene and 1,5-dihydroxynaphthalene as guest molecules was investigated by NMR titration experiments and molecular modelling.

**Figure 3:**
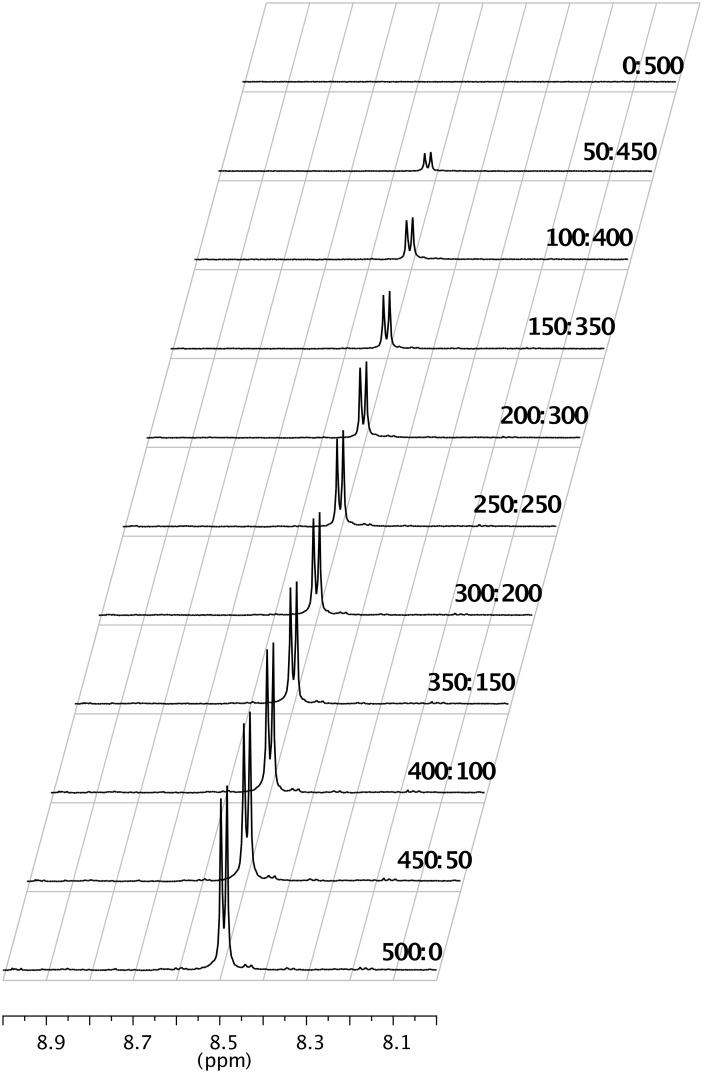
Chemical shift changes of the reference signal (belonging to the more deshielded protons of the *p*-phenylene units of cryptand **2**) in correlation with the changes of the cryptand:1,5-dihydroxynaphthalene ratios.

The spectra recorded during the NMR titrations of cryptand **2** with increasing amounts of the named guests (ratio 1:9 to 9:1), revealed only one set of signals. However, the resonances continuously changed positions during variation of the host:guest ratios thus proving that the formation of the host–guest complexes is a fast, dynamic process. The changes of the chemical shift for the signal belonging to the more deshielded signal of the *p*-phenylene units (doublet at δ 8.49 ppm, marked with a black circle in Figure 2) of cryptand **2** upon titration with 1,5-dihydroxynaphthalene at different host:guest ratios are shown in Figure 3.

Next the host–guest interactions were investigated by global non-linear regression analysis using the free tool that is available at http://supramolecular.org [37–39]. The primary data, δ values at different host:guest ratios, were introduced in the program available on this site and the experimental curves for different binding models were compared with the theoretical ones, selecting the experimental model which highly fitted the theoretical one. Global non-linear regression analysis of the ^1^H NMR titration data indicated the formation of 1:1 stoichiometric complexes. The association constants for the complexation of **2** with anthracene, pyrene and 1,5-dihydroxynaphthalene are 472.81 M^−1^ ± 4.9882, 55.22 M^−1^ ± 2.7771 and 21.34 M^−1^ ± 2.7909, respectively (M^−1^ = L/mol). The details of these experiments are given in Supporting Information File 1.

**Figure 4:**
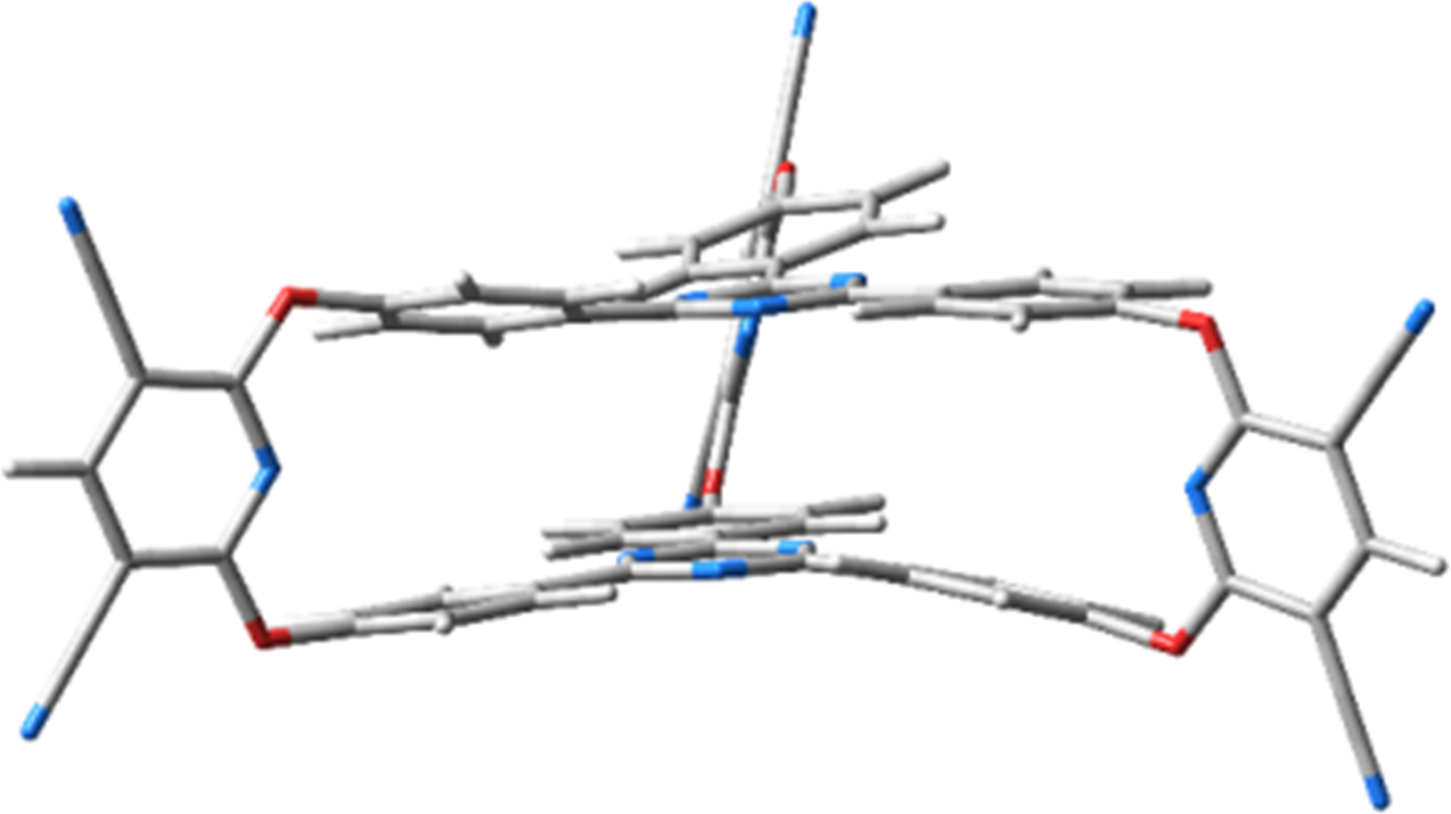
The equilibrium geometry structure of cryptand **2** having 2,4,6-triphenyl-1,3,5-triazine caps.

The first step in the molecular modelling investigations was the optimization of the equilibrium geometry of the molecular structure of cryptand **2**, the result being shown in Figure 4.

**Figure 5:**
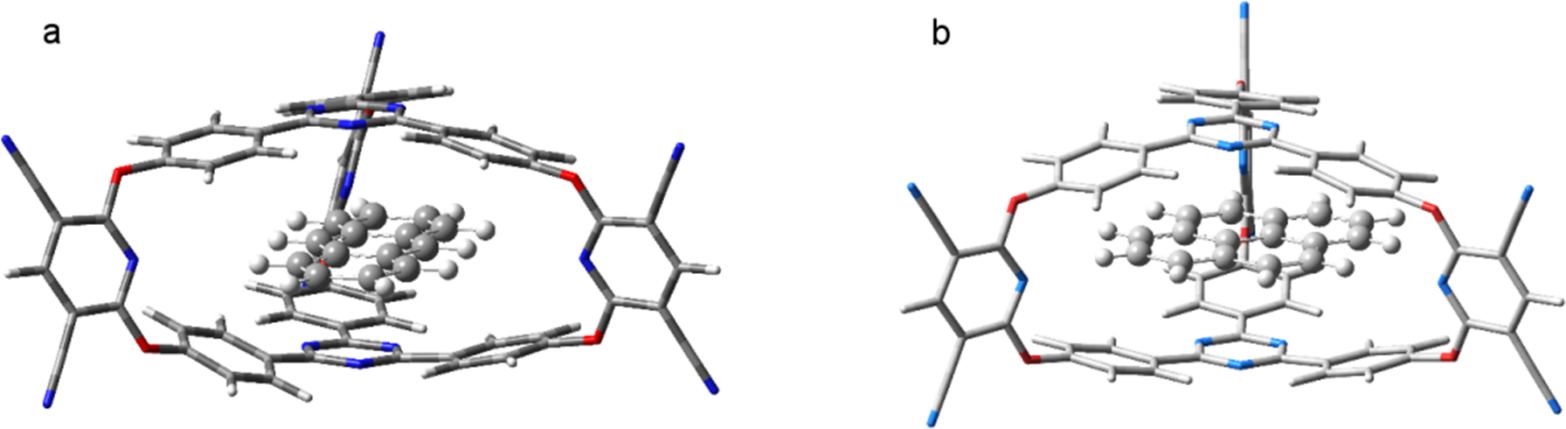
The equilibrium geometry structures of the cryptand–anthracene (a) and cryptand–pyrene (b) host–guest complexes.

The geometry structure of cryptand **2** using the M11/Def2-TZVP calculation reveals a more regular structure than for the previously studied cryptand based on 1,3,5-triphenylbenzene caps [32]. In **2**, the benzene rings are not distorted, the phenyl groups being almost coplanar with the central 1,3,5-triazine unit and the planes of the two caps are parallel. The central triazine rings in **2** are close to each other, the distance between the heterocycles being 3.63 Å. This value is smaller compared to the distance (3.72 Å) measured between the benzene molecules in their sandwich configuration [40], but it is larger than the distance (3.15 Å) determined for the triazine dimer [41]. As next step, the polycyclic aromatic hydrocarbons anthracene and pyrene were intercalated between the caps of the cryptand. The optimized geometry structures of these host–guest complexes were obtained using the same method/basis set calculation setup and the optimized structures of the cryptand–anthracene and cryptand–pyrene complexes are presented in Figure 5.

**Figure 6:**
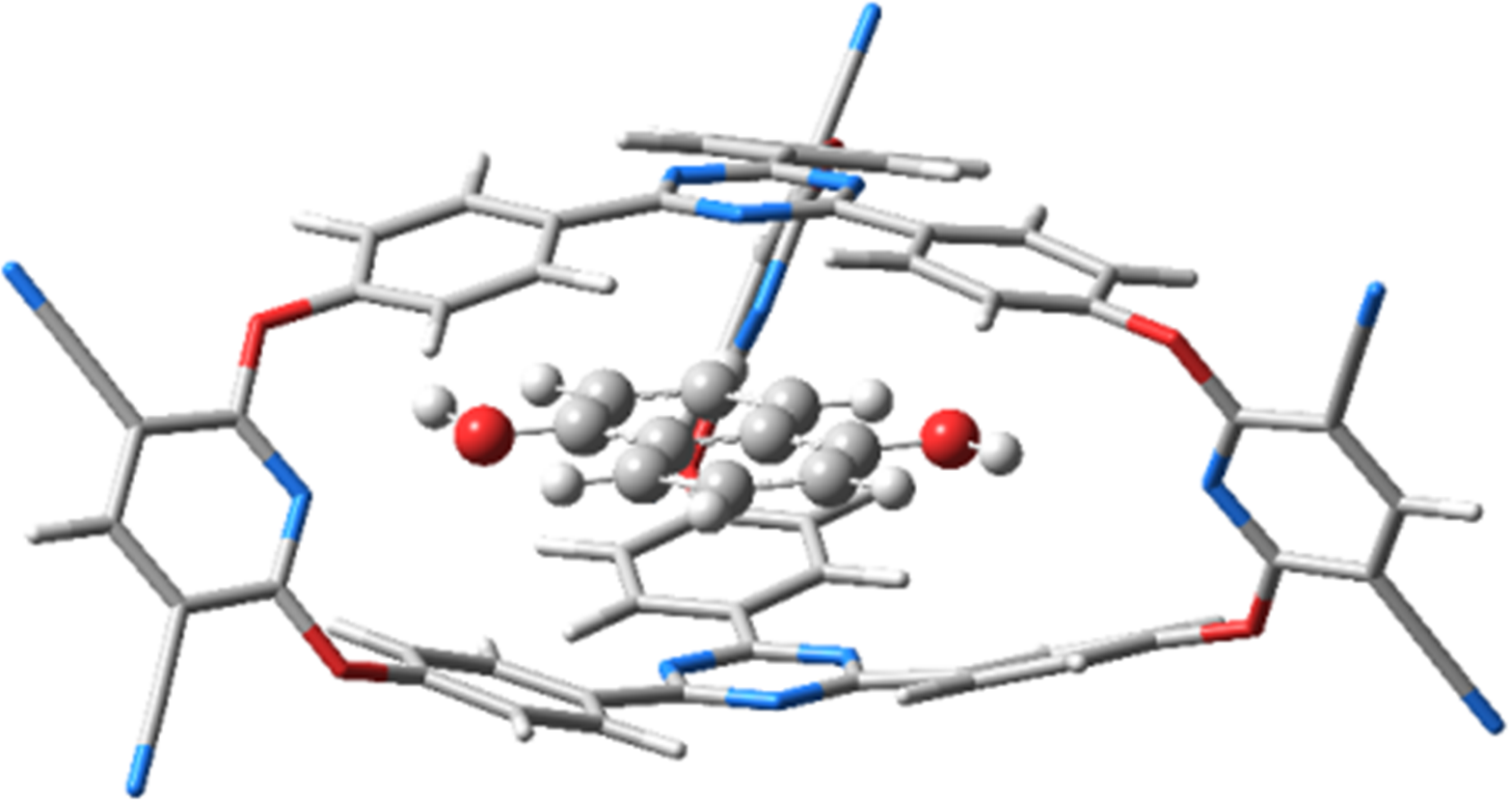
The equilibrium geometry structure of the cryptand **2**–1,5-dihydroxynaphthalene host–guest complex.

The intermolecular interaction between anthracene and cryptand **2** is −30.64 kcal/mol and −29.70 kcal/mol between pyrene and the cryptand. The deformation potential of **2** in the case of the cryptand–anthracene complex is +11.44 kcal/mol and +17.41 kcal/mol for the corresponding pyrene complex. Since the anthracene molecule enters the cavity of the cryptand with its longitudinal direction the deformation energy of the cryptand becomes lower and the energy balance between the intermolecular bounding and the energy loss due to the deformation becomes more favorable for anthracene than for pyrene. At the same time, the stacking distances between the anthracene and the caps of cryptand **2** are 3.08 Å and 3.15 Å, respectively, while between pyrene and the caps of cryptand **2** they were 3.27 Å and 3.21 Å, respectively. Since the 3,5-dicyanopyridine fragment contains hydrogen-bond acceptor nitrogen atoms it might be possible to select a guest system which besides the stacking interaction could establish extra hydrogen bonds to enhance the complex stability. Accordingly, 1,5-dihydroxynaphthalene was chosen as another candidate for intercalation in the cryptand system. The pyrene molecule was replaced by 1,5-dihydroxynaphthalene in such way that its OH fragment arrived near the N atom of the 3,5-dicyanopyridine fragment. However, at the end of the energy optimization, the O–H···N fragment did not preserve its hydrogen-bond arrangement, as the aromatic fragment rather preferred the so-called “antiparallel-displaced” configuration [40–41] (see Figure 6). The intermolecular interaction energy between 1,5-dihydroxynaphthalene and cryptand **2** is −29.28 kcal/mol, while the deformation potential of the cryptand is +11.85 kcal/mol giving an overall binding energy of −17.43 kcal/mol. The stacking distances between the aromatic ring of the 1,5-dihydroxynaphthalene molecule and the cryptand caps are 3.17 Å and 3.20 Å, respectively.

Due to the discrepancies between the experimental (the order of the association constants) and theoretical (the magnitude of the binding energies) results, the intermolecular interaction energies were recomputed considering solvent effects induced by the DMSO solvent environment. Accordingly, the intermolecular interaction energies for the different host–guest complexations were −26.30 kcal/mol for cryptand–anthracene, −22.64 kcal/mol for cryptand–pyrene and −21.44 for cryptand–1,5-dihydroxynaphthalene cases, respectively.

The higher values obtained by the NMR titration experiments for the complexation of the unsubstituted polyaromatic guests anthracene and pyrene in comparison with 1,5-dihydroxynaphthalene are somewhat in contradiction with the molecular modelling results obtained in the gas phase. However, the influence of DMSO as the solvent on the dihydroxylated guest is more pronounced than on the aromatic hydrocarbons. This stronger solvent–guest interaction in the case of the dihydroxylated guest leads to a weaker host–guest interaction and consequently to a lower association constant. This may explain the different results obtained from the experimental and theoretical data. Indeed, the recalculation including the influence of the solvent environment afforded more realistic results for the complexation of the three guest molecules. Namely, the strongest binding was found for anthracene, followed by pyrene, whereas the weakest host–guest interaction was calculated for the cryptand–1,5-dihydroxynaphthalene complex.

**Figure 7:**
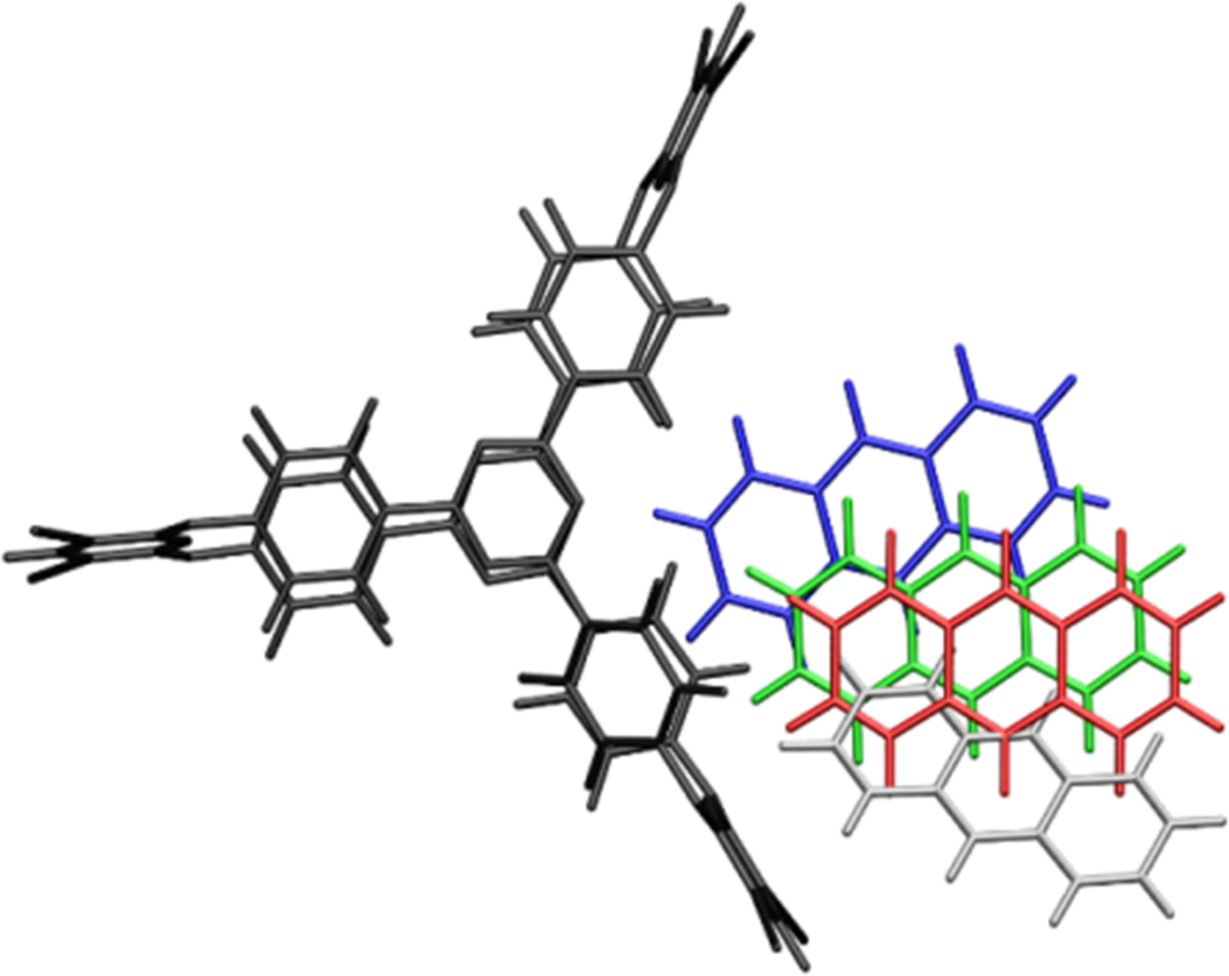
The inclusion dynamics of the anthracene in the cavity of the cryptand for different constrained distances (blue < green < red < grey).

We consider that another major impediment to form full complexation, i.e., the guest molecule enters entirely inside the cryptand’s cavity, is the fitting dynamics. This process might have a strong entropic character and the energetic contribution can also give valuable information about the efficiency of the fitting. Accordingly, a further constrained geometry optimization was performed for the cryptand–anthracene case, where the distance between the 3,5-dicyanopyridine fragment of **2** and the carbon atom of the anthracene was kept constant for each optimization case. The inclusion dynamics of the anthracene in the cavity of **2** for different constrained distances is compiled in Figure 7, where the anthracene molecule is shown with different colors depending on the constrained distance, i.e., the shortest distance is colored with blue, followed by green, red and grey. The intermolecular energies between the cryptand and the anthracene depending on the constrained distance are: −6.00 kcal/mol (blue), −3.74 kcal/mol (green), −2.63 kcal/mol (red) and −0.76 kcal/mol (grey). This decrease of the binding energy tells us that even for the anthracene the fitting dynamics is not a straightforward process.

## Conclusion

The 1,3,5-triazine units in cryptand **2** exhibit a slight steric hindrance with the phenyl groups located at positions 2, 4 and 6 and thus, the reference triphenyltriazine aromatic platforms can adopt a planar structure which is favorable for the binding of aromatic guests such as anthracene, pyrene or 1,5-dihydroxynaphthalene. The higher affinity for anthracene and pyrene than for the dihydroxylated aromatic guest is explained by the influence of the polar solvent (DMSO). Molecular modelling revealed the preference for aryl–aryl stacking instead of a hydrogen bond between the donor OH groups of the guest and the acceptor pyridine rings of the host. The association constants (determined by NMR titrations) ranged between 21.34–472.81 M^−1^ (L/mol). A simple energetic study revealed that the fitting dynamics of the guest molecules inside the cryptand cavity is not a straightforward process, the binding energies between the host and guest molecules should overcome the magnitude of the deformation potential during the inclusion process.

## Experimental

**Computational details:** The equilibrium geometries and the intermolecular interaction energies for different host–guest assemblies between the cryptand **2** and pyrene, anthracene and 1,5-dihydroxynaphthalene have been obtained at density functional theory level, using the M11 [42] exchange-correlation functional and the def2-TZVP [43] basis set implemented in the Gaussian 09 program package [44]. In order to take into account the solvent effect induced by the DMSO solvent environment, the equilibrium geometries of the host–guest constituents were reoptimized considering the PCM (Polarizable Continuum Model) solvent model [45]. By construction, M11 was parameterized to mimic short and intermediate-range dispersion effects; therefore, it can be considered suitable for describing van der Waals complexes near their equilibrium geometries [46]. The so-called basis set superposition error (BSSE) was not taken into account for the geometry optimization and intermolecular energy calculation, because this correction was included in the parametrization procedure of the XC functional [42].

**General data:** ^1^H NMR (300 MHz) and ^13^C NMR (75 MHz) spectra were recorded in CDCl_3_ at rt at 300 MHz using the residual solvent signal as reference. Atmospheric pressure chemical ionization mass spectra (APCIMS, negative ion mode) were recorded on LTQ ORBITRAP XL spectrometer using external mass calibration. Melting points were measured with a routine apparatus. The triazine **3** was prepared according to a procedure described in the literature [36], while the other reagents were commercially available and used without further purification. Thin-layer chromatography (TLC) was conducted on silica gel 60 F254 TLC plates. Solvents were dried and distilled under argon using standard procedures.

Procedure for the synthesis of **2** (analogous to the method described for **1** in [32]): Triphenol **3** (0.10 g, 0.28 mmol), 2,6-dichloro-3,5-dicyanopyridine (0.083 g, 0.42 mmol) and anhydrous NEt_3_ (0.12 mL, 0.84 mmol) were dissolved at rt under an Ar atmosphere under stirring in 10 mL anhydrous DMSO. The temperature was raised to 80 °C and the vigorous stirring was continued at this temperature overnight (12 h). The reaction mixture was then cooled to rt and partitioned between ethyl acetate (50 mL) and 1 M HCl solution (40 mL). The layers were separated, and the aqueous phase was extracted 3 times with ethyl acetate (3 × 20 mL). The combined organic layers were washed with brine (50 mL), dried over Na_2_SO_4_, filtered and concentrated in vacuum. The resulting residue was purified by column chromatography on silica gel (eluent acetone/pentane 1:1.5) to give pure cryptand **2** as a yellow solid (0.128 g, 42%).

**12,14,32,34,48,50-Hexacyano-10,16,30,36,46,52-hexaoxa-2,4,22,24,41,57,60,65,70-nonaazatridecacyclo[23.15.2.^6,9^2.^17,20^2.^26,29^2.^37,40^2.^42,45^2.^53,56^1.^1,5^1.^11,15^1.^21,25^1.^31,35^1.^47,51^]doheptaconta-1,3,5(41),6,8,11,13,15(65),17,19,21,23,25(57),26,28,31,33, 35(60),37,39,42,44,47,49,51(70),53,55,58,61,63,66,68,71-tritricontaene (2).** Yellow solid (yield 42%); mp >360 °C; Anal. calcd for C_63_H_27_N_15_O_6_: C, 69.42; H, 2.50; N 19.28; found: C, 69.59; H, 2.61; N, 19.06 (%); ^1^H NMR (400 MHz, DMSO-*d*_6_) δ 7.23 (d, *J* = 8.8 Hz, 12H, 8-H, 18-H, 28-H, 38-H, 44-H, 54-H, 59-H, 61-H, 64-H, 66-H, 69-H, 71-H), 8.49 (d, *J* = 8.8 Hz, 12H, 7-H, 19-H, 27-H, 39-H, 43-H, 55-H, 58-H, 62-H, 63-H, 67-H, 68-H, 72-H), 9.16 ppm (s, 3H, 13-H, 33-H, 49-H); ^13^C NMR (100 MHz, DMSO-*d*_6_) δ 89.82 (12-C, 14-C, 32-C, 34-C, 48-C, 50-C), 113.58 (8-C, 18-C, 28-C, 38-C, 44-C, 54-C, 59-C, 61-C, 64-C, 66-C, 69-C, 71-C), 122.46 (CN), 130.05 (6-C, 20-C, 26-C, 40-C, 42-C, 56-C), 132.95 (7-C, 19-C, 27-C, 39-C, 43-C, 55-C, 58-C, 62-C,63-C, 67-C, 68-C, 72-C), 151.66 (1-C, 3-C, 5-C, 21-C, 23-C, 25-C), 155.08 (13-C, 33-C, 49-C), 164.61 (9-C, 17-C, 29-C, 37-C, 45-C, 53-C), 169.87 (11-C, 15-C, 31-C, 35-C, 47-C, 51-C); MS–APCI^+^ (*m*/*z*): [M + H]^+^ 1090.3 (calcd 1090.2).

## Supporting Information

File 1 Complexation experiments and atomic coordinates.
