# Supplementary material for: A three-armed cryptand with triazine and pyridine units: synthesis, structure and complexation with polycyclic aromatic compounds
Source: Beilstein J Org Chem. 2018 Jun 6;14:1370–7. doi: 10.3762/bjoc.14.115 (PMC6009098; doi:10.3762/bjoc.14.115)
Supplement: File 1 — Complexation experiments and atomic coordinates. [file Beilstein_J_Org_Chem-14-1370-s001.pdf]

Supporting Information

for

A three-armed cryptand with triazine and pyridine units: synthesis, structure and complexation with polycyclic aromatic compounds

Claudia Lar[1,2], Adrian Woiczechowski-Pop[1], Attila Bende[2], Ioana Georgeta Grosu[2], Natalia Miklášová[3], Elena Bogdan[1], Niculina Daniela Hădade[1], Anamaria Terec*[1] and Ion Grosu*[1]

Address: [1]Babes-Bolyai University, Faculty of Chemistry and Chemical Engineering, Department of Chemistry and SOOMCC, Cluj-Napoca, 11 Arany Janos str., 400028, Cluj-Napoca, Romania, [2]National Institute for Research and Development of Isotopic and Molecular Technologies, 67-103 Donath str., RO-400293, Cluj-Napoca, Romania and [3]Department of Chemical Theory of Drugs, Faculty of Pharmacy, Comenius University in Bratislava, Kalinčiakova 8, 83104, Bratislava, Slovakia

Email: Terec Anamaria - asuciu@chem.ubbcluj.ro; Grosu Ion -igrosu@chem.ubbcluj.ro

* Corresponding author

Complexation experiments and atomic coordinates

Table of contents

1. Description of complexation experiments S2
2. References S4
3. Atomic coordinates for the calculated structures S5

Description of complexation experiments

The stoichiometry and association constants for the formation of host–guest complexes between cryptand **2** and different guests anthracene (G1), pyrene (G2) and 1,5-dihydroxynaphthalene (G3) were determined by global non-linear regression analysis[1] of the ^{1}H NMR titration data. In each case nine NMR samples at various host:guest ratios (Tables S1–S3) were prepared using 3 mM stock solutions of **2** (host, 3 mM)in DMSO-d_6 and of the target guests G1–G3 at a final concentration of 3 mM (host [H] + guest [G]).

The changes in the chemical shifts of the signal at 8.4922 ppm of cryptand **2** for increasing amounts of guest are indicated in Tables S1–S3.

Table S1: Experimental data for the NMR titration of **2** with anthracene (G1).

Cryptand H (μL)	Anthracene G1 (μL)	[H] (mM)	[G1] (mM)	δ(ppm)
50	450	0.003	0.027	8.5055
100	400	0.006	0.024	8.5046
150	350	0.009	0.021	8.503
200	300	0.012	0.018	8.5006
250	250	0.015	0.015	8.4991
300	200	0.018	0.012	8.4973
350	150	0.021	0.009	8.4953
400	100	0.024	0.006	8.493
450	50	0.027	0.003	8.4915

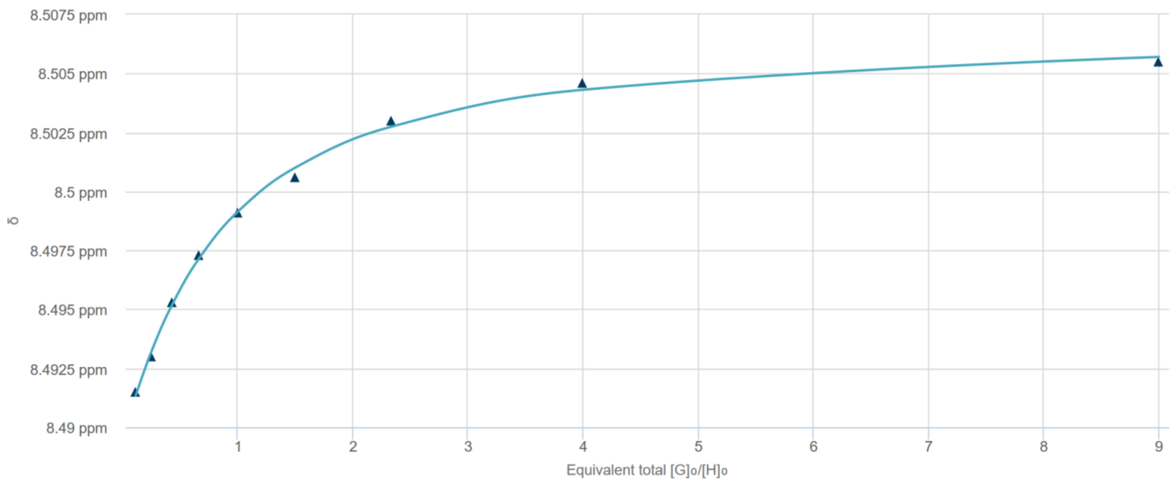

Figure S1: Plot of the data for the titration **2** with anthracene (G1) showing 1:1 stoichiometry.

Table S2: Experimental data for the NMR titration of **2** with pyrene (G2).

**Cryptand H (μL)**	**Pyrene G2 (μL)**	**[H] (mM)**	**[G2] (mM)**	**δ(ppm)**
50	450	0.003	0.027	8.5037
100	400	0.006	0.024	8.5029
150	350	0.009	0.021	8.5018
200	300	0.012	0.018	8.5004
250	250	0.015	0.015	8.4991
300	200	0.018	0.012	8.4975
350	150	0.021	0.009	8.4963
400	100	0.024	0.006	8.4947
450	50	0.027	0.003	8.4934

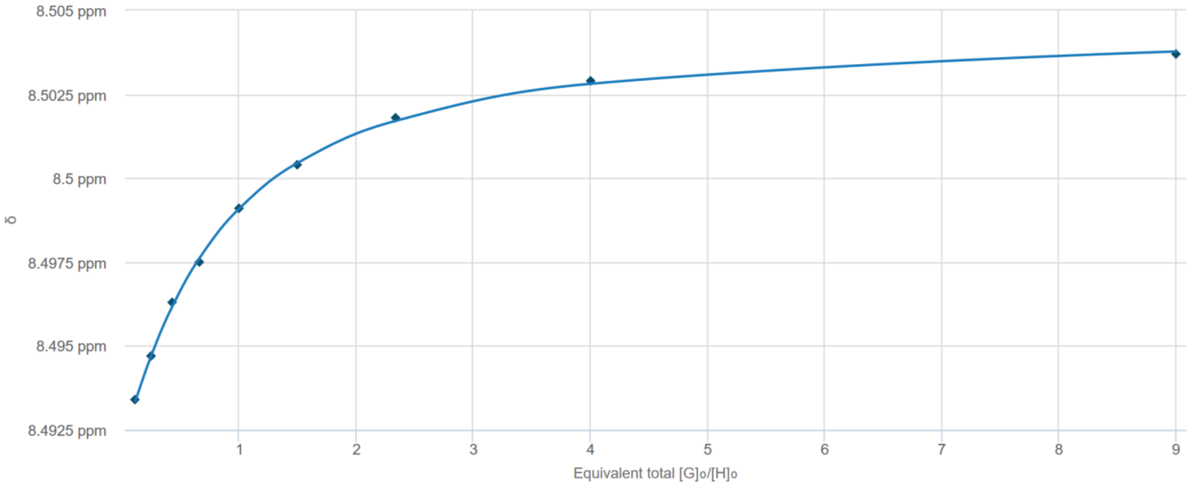

Figure S2: Plot of the data for the titration **2** with pyrene (G2) showing 1:1 stoichiometry.

Table S3: Experimental data for the NMR titration of **2** with 1,5-dihydroxynaphthalene (G3).

**Cryptand H (μL)**	**1,5-dihydroxynaphthalene G3 (μL)**	**[H] (mM)**	**[G3] (mM)**	**δ(ppm)**
50	450	0.003	0.027	8.506
100	400	0.006	0.024	8.504
150	350	0.009	0.021	8.5025
200	300	0.012	0.018	8.501
250	250	0.015	0.015	8.499
300	200	0.018	0.012	8.4975
350	150	0.021	0.009	8.496
400	100	0.024	0.006	8.494
450	50	0.027	0.003	8.4925

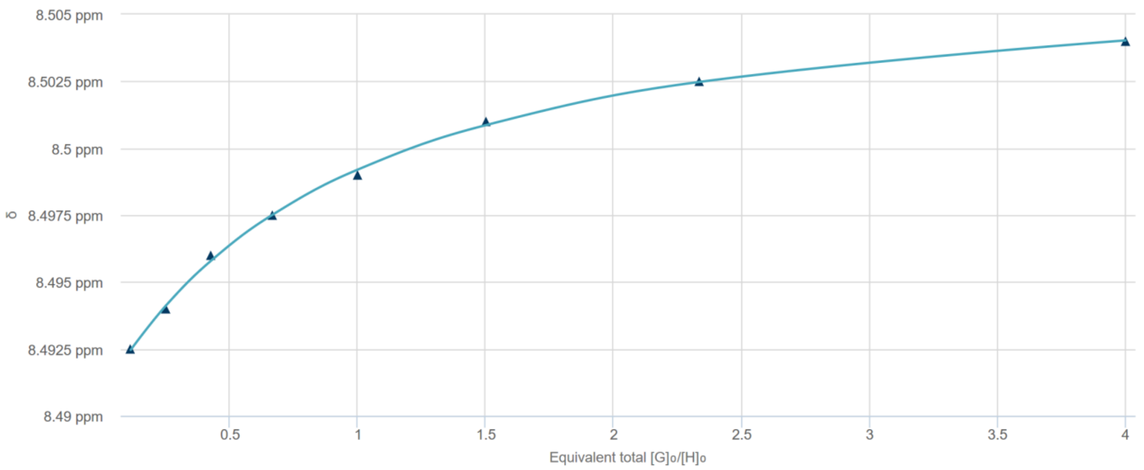

Figure S3: Plot of the data for the titration **2** with 1,5-dihydroxynaphthalene (G3) showing 1:1 stoichiometry.

The curves were best fitted for the formation of 1:1 stoichiometric complexes in all cases. The values of the association constants calculated by global non-linear regression analysis from ^{1}H NMR titration data are presented in Table S4.

Table S4: Association constants (M^{-1}) for complexation of **2** with anthracene (G1), pyrene (G2) and 1,5-dihydroxynaphthalene (G3).

Guest	K (M^{-1})
anthracene (G1)	472.81 ± 4.9882
pyrene (G2)	55.22 ± 2.7771
1,5-dihydroxynaphthalene (G3)	21.34 ± 27909

References

1) Thordarson, P. *Chem. Soc. Rev*. **2011**, *40*, 1305-1323; (b). Hibbert, D. B; Thordarson, P. Chem. Commun. **2016**, 53, 12792-12805.

Atoms coordinates for the molecular modeling results

<u>In gas phase:</u>

Cryptand **2**:

111

N	0.85763	0.28884	1.56846
C	-0.14292	1.15112	1.71543
N	-1.40998	0.79001	1.87934
C	-1.64505	-0.51616	1.93297
N	-0.69403	-1.44199	1.87390
C	0.54566	-0.99743	1.70035
C	0.18376	2.59793	1.77813
C	-0.77863	3.50436	2.20655
C	-0.45525	4.84104	2.37187
C	0.83362	5.25062	2.09957
C	1.80445	4.37152	1.65156
C	1.47362	3.03929	1.49363
H	-1.77813	3.14441	2.42866
H	-1.18433	5.56506	2.72276
H	2.80276	4.73728	1.43054
H	2.21323	2.31935	1.15387
C	-3.05174	-0.96186	2.07779
C	-3.35511	-2.31995	2.08874
C	-4.67121	-2.73462	2.14378
C	-5.66659	-1.77769	2.17409
C	-5.39222	-0.42620	2.21407
C	-4.07070	-0.02020	2.16546
H	-2.54509	-3.03990	2.02910
H	-4.93837	-3.78696	2.11335
H	-6.20996	0.28742	2.24815
H	-3.81292	1.03415	2.16822
C	1.65756	-1.98119	1.73696
C	2.96557	-1.56457	1.49878
C	4.01725	-2.44673	1.64524
C	3.74901	-3.74733	2.04100
C	2.46050	-4.18923	2.26306
C	1.41091	-3.29749	2.10742
H	3.14460	-0.53296	1.20897
H	5.04081	-2.13332	1.46643
H	2.29460	-5.21586	2.57454
H	0.38819	-3.60769	2.29682
O	1.15035	6.59838	2.31156
C	1.33173	7.36882	1.23540
N	1.23848	6.81778	0.05070
C	1.39378	7.53679	-1.03500
O	1.29614	6.92105	-2.21873
C	1.60354	8.73440	1.40940
C	1.76429	9.49637	0.26110
C	1.66122	8.91308	-0.99370
C	1.69944	9.30431	2.71630
N	1.77790	9.77239	3.75781
H	1.97302	10.55903	0.34525
C	1.82523	9.66649	-2.19648
N	1.95979	10.27966	-3.15336
O	-7.00322	-2.18834	2.08360
C	-7.42373	-2.47053	0.84437
N	-6.57430	-2.33028	-0.14666

C	-6.93415	-2.60724	-1.37492
O	-6.03437	-2.47925	-2.35279
C	-8.74367	-2.90198	0.64955
C	-9.12645	-3.18992	-0.65277
C	-8.22614	-3.05029	-1.69873
C	-9.64418	-3.03655	1.75069
N	-10.37635	-3.14810	2.62319
H	-10.13831	-3.52957	-0.85427
C	-8.58460	-3.34697	-3.04993
N	-8.88473	-3.58991	-4.12738
O	4.79193	-4.65031	2.28008
C	5.60282	-4.97466	1.26910
N	5.36325	-4.46465	0.08852
C	6.11599	-4.76060	-0.94180
O	5.81841	-4.19630	-2.11898
C	6.66949	-5.85712	1.49821
C	7.46968	-6.17335	0.40909
C	7.21068	-5.63108	-0.84255
C	6.90763	-6.40399	2.79678
N	7.10711	-6.84947	3.83199
H	8.30717	-6.85318	0.53714
C	8.01734	-5.93568	-1.98218
N	8.67096	-6.18726	-2.88744
N	1.61125	0.57995	-1.89184
C	0.53713	1.35722	-1.81135
N	-0.70290	0.89568	-1.67785
C	-0.83923	-0.42668	-1.68602
N	0.18365	-1.27256	-1.74539
C	1.39194	-0.72884	-1.84609
C	0.73006	2.82365	-1.93257
C	-0.36057	3.68237	-1.82798
C	-0.18443	5.04742	-1.94879
C	1.09023	5.53540	-2.16621
C	2.18437	4.70506	-2.29625
C	1.99745	3.33868	-2.17913
H	-1.34411	3.26176	-1.64304
H	-1.01517	5.73960	-1.84832
H	3.16575	5.13440	-2.47510
H	2.83410	2.65368	-2.27303
C	-2.21461	-0.98879	-1.71763
C	-2.40526	-2.32710	-2.04274
C	-3.68426	-2.82968	-2.21676
C	-4.76120	-1.98180	-2.05334
C	-4.59880	-0.65695	-1.68444
C	-3.31827	-0.16132	-1.52547
H	-1.53715	-2.96185	-2.18961
H	-3.85551	-3.86182	-2.50629
H	-5.47015	-0.02375	-1.54584
H	-3.15456	0.88100	-1.26702
C	2.55735	-1.64033	-1.95412
C	3.83886	-1.12590	-2.10444
C	4.92939	-1.97826	-2.18089
C	4.70989	-3.33812	-2.11526
C	3.44005	-3.87425	-2.00126
C	2.36215	-3.01853	-1.91125
H	3.97197	-0.04952	-2.14932
H	5.94332	-1.60210	-2.27778
H	3.31438	-4.95143	-1.94458
H	1.35394	-3.40256	-1.79375

Complex cryptand **2** – anthracene:

135

C	-0.99076	0.84470	-0.00833
C	0.18745	0.10654	0.01508
C	1.47434	0.73000	0.02673
C	2.60655	-0.01244	0.01115
C	2.53838	-1.43635	0.00057
C	1.33834	-2.06746	0.01281
C	0.12030	-1.31962	0.01141
C	-1.12041	-1.94418	-0.01077
C	-2.29820	-1.20523	-0.05056
C	-3.57688	-1.83949	-0.09387
C	-4.71339	-1.10116	-0.14648
C	-4.64765	0.32369	-0.16779
C	-3.45072	0.96018	-0.12506
C	-2.23329	0.21785	-0.05342
H	-0.94399	1.93437	-0.00511
H	1.52212	1.81905	0.03942
H	3.58378	0.47203	0.01850
H	3.46340	-2.00872	-0.00599
H	1.27076	-3.15446	0.01176
H	-1.17220	-3.03693	-0.00813
H	-3.60707	-2.92886	-0.08325
H	-5.68344	-1.59036	-0.18057
H	-5.56879	0.89829	-0.23180
H	-3.39042	2.04879	-0.15834
N	1.04184	-0.96966	3.18481
C	1.54285	0.25820	3.14154
N	0.80029	1.36045	3.09973
C	-0.51390	1.17844	3.08530
N	-1.09750	-0.01431	3.12954
C	-0.28414	-1.06178	3.15601
C	3.01516	0.40109	3.01193
C	3.56686	1.64156	2.70617
C	4.90655	1.75271	2.38300
C	5.68308	0.60656	2.37849
C	5.16653	-0.62948	2.71672
C	3.82290	-0.72851	3.03120
H	2.91867	2.51249	2.69157
H	5.35173	2.71114	2.13289
H	5.82303	-1.49415	2.73039
H	3.37497	-1.69005	3.26243
C	-1.38098	2.37235	2.91164
C	-2.74769	2.21192	2.70973
C	-3.54866	3.30701	2.44246
C	-2.96520	4.55902	2.38638
C	-1.61262	4.74740	2.60175
C	-0.81922	3.64379	2.86099
H	-3.16902	1.21169	2.73703
H	-4.61590	3.19500	2.27222
H	-1.20313	5.75232	2.57262
H	0.24848	3.75347	3.02343
C	-0.89201	-2.41078	3.03336
C	-0.08709	-3.51064	2.75051
C	-0.65737	-4.73398	2.44722
C	-2.03806	-4.83940	2.44957
C	-2.85492	-3.77040	2.76185
C	-2.27430	-2.54831	3.04642
H	0.99113	-3.37962	2.73784

H	-0.04773	-5.60267	2.21489
H	-3.93197	-3.90706	2.77309
H	-2.88977	-1.67613	3.24561
O	7.05232	0.67972	2.15238
C	7.58885	1.13471	1.01933
N	6.85508	1.30785	-0.06392
C	7.44969	1.73882	-1.16019
O	6.76054	1.94316	-2.28211
C	8.97134	1.38153	1.06320
C	9.58185	1.83668	-0.09139
C	8.82359	2.02690	-1.23172
C	9.70646	1.16881	2.27013
N	10.31146	1.00395	3.22745
H	10.64804	2.04307	-0.10200
C	9.40310	2.49541	-2.45138
N	9.88653	2.87243	-3.41781
O	-3.75459	5.69351	2.21933
C	-4.47559	5.91498	1.11973
N	-4.29636	5.18807	0.03437
C	-4.99831	5.47622	-1.04467
O	-4.84961	4.77806	-2.17149
C	-5.39525	6.97486	1.19148
C	-6.14116	7.25597	0.06107
C	-5.95336	6.50574	-1.08617
C	-5.54522	7.72455	2.39883
N	-5.67948	8.33538	3.35718
H	-6.86682	8.06396	0.07257
C	-6.69234	6.76204	-2.28245
N	-7.29434	6.98118	-3.23083
O	-2.65383	-6.07212	2.26077
C	-2.56152	-6.78020	1.13612
N	-2.07873	-6.24729	0.02851
C	-2.03243	-6.99835	-1.05609
O	-1.54150	-6.52799	-2.20198
C	-3.03498	-8.10073	1.21605
C	-2.97984	-8.87652	0.07281
C	-2.47086	-8.33360	-1.09202
C	-3.54934	-8.61236	2.44779
N	-3.96584	-9.04147	3.42375
H	-3.33313	-9.90334	0.09018
C	-2.39206	-9.08937	-2.30255
N	-2.33857	-9.71168	-3.26164
N	1.11296	-0.84746	-3.12993
C	1.40297	0.44554	-3.08604
N	0.48685	1.40843	-3.05238
C	-0.77902	1.01189	-3.07811
N	-1.15827	-0.26077	-3.13545
C	-0.18056	-1.15809	-3.12455
C	2.83113	0.83971	-2.96647
C	3.15915	2.17207	-2.74998
C	4.46715	2.53548	-2.48270
C	5.43374	1.55003	-2.43909
C	5.13676	0.21900	-2.67228
C	3.82536	-0.13255	-2.93660
H	2.36980	2.91679	-2.77787
H	4.75132	3.57076	-2.32138
H	5.93080	-0.52192	-2.65120
H	3.54843	-1.17064	-3.09489
C	-1.83750	2.04253	-2.93438
C	-3.16244	1.74008	-3.21574

C	-4.15767	2.66689	-2.95563
C	-3.81346	3.87650	-2.38390
C	-2.49558	4.21249	-2.11828
C	-1.50982	3.28924	-2.40845
H	-3.40839	0.75897	-3.61107
H	-5.20182	2.45798	-3.16768
H	-2.25177	5.17701	-1.68603
H	-0.46633	3.51222	-2.20489
C	-0.54919	-2.59023	-2.98971
C	0.44674	-3.53957	-2.79789
C	0.11340	-4.84941	-2.49966
C	-1.22179	-5.18732	-2.39844
C	-2.23282	-4.26608	-2.60856
C	-1.88835	-2.96069	-2.90507
H	1.48520	-3.22784	-2.85810
H	0.87067	-5.61407	-2.35516
H	-3.27139	-4.57739	-2.53832
H	-2.65304	-2.20011	-3.03427

Complex cryptand **2** – pyrene:

137

N	0.2577690000	-1.2736520000	-3.2455290000
C	-1.0253070000	-0.9281430000	-3.2386600000
N	-1.4516770000	0.3296250000	-3.2402560000
C	-0.5122270000	1.2687110000	-3.2203760000
N	0.7898730000	1.0098410000	-3.2217260000
C	1.1329250000	-0.2740780000	-3.2265650000
C	-2.0417030000	-2.0062380000	-3.1156790000
C	-3.3771980000	-1.6683050000	-2.9334470000
C	-4.3060640000	-2.6404630000	-2.6068470000
C	-3.8792440000	-3.9470070000	-2.4728790000
C	-2.5666740000	-4.3183570000	-2.7026550000
C	-1.6467240000	-3.3366220000	-3.0230920000
H	-3.6682880000	-0.6250620000	-3.0086870000
H	-5.3546410000	-2.3992570000	-2.4587890000
H	-2.2836770000	-5.3635730000	-2.6256020000
H	-0.5988160000	-3.5823460000	-3.1649330000
C	-0.9482350000	2.6837420000	-3.0870330000
C	-0.0074160000	3.6993220000	-2.9408750000
C	-0.4082650000	4.9834380000	-2.6219530000
C	-1.7594520000	5.2324030000	-2.4547490000
C	-2.7149080000	4.2551010000	-2.6471180000
C	-2.3005650000	2.9729700000	-2.9626160000
H	1.0455490000	3.4538560000	-3.0401680000
H	0.3079640000	5.7899170000	-2.4959400000
H	-3.7664460000	4.5060240000	-2.5456760000
H	-3.0201250000	2.1680760000	-3.0766280000
C	2.5773060000	-0.6068200000	-3.1148200000
C	2.9856640000	-1.9328380000	-3.0269180000
C	4.3101370000	-2.2433050000	-2.7715820000
C	5.2137280000	-1.2100660000	-2.6037180000
C	4.8389300000	0.1159560000	-2.7178440000
C	3.5125090000	0.4123710000	-2.9746100000
H	2.2423750000	-2.7170460000	-3.1340740000
H	4.6516360000	-3.2724270000	-2.7104800000
H	5.5880660000	0.8955310000	-2.6172040000
H	3.1762380000	1.4425940000	-3.0390360000
O	-4.8264620000	-4.9385870000	-2.2355020000
C	-5.1937480000	-5.3525710000	-1.0234310000

N	-4.6484890000	-4.8627110000	0.0782110000
C	-5.0574960000	-5.3476290000	1.2385490000
O	-4.5526190000	-4.9316450000	2.3981360000
C	-6.1897150000	-6.3445710000	-1.0214810000
C	-6.6100550000	-6.8374890000	0.1984360000
C	-6.0439670000	-6.3437910000	1.3577540000
C	-6.7372440000	-6.8219650000	-2.2524240000
N	-7.1905270000	-7.2201090000	-3.2250950000
H	-7.3757620000	-7.6062860000	0.2458110000
C	-6.4374580000	-6.8198210000	2.6465610000
N	-6.7712700000	-7.2122250000	3.6685370000
O	-2.1982250000	6.5314570000	-2.2154250000
C	-2.1438480000	7.1325550000	-1.0280300000
N	-1.7457750000	6.4938800000	0.0593450000
C	-1.7149900000	7.1673850000	1.1968620000
O	-1.2964430000	6.6142220000	2.3341390000
C	-2.5482280000	8.4794770000	-1.0318950000
C	-2.5132180000	9.1688050000	0.1648110000
C	-2.0898080000	8.5181370000	1.3077060000
C	-2.9737870000	9.1025510000	-2.2454830000
N	-3.3187080000	9.6211670000	-3.2055660000
H	-2.8128670000	10.2119250000	0.2064340000
C	-2.0285130000	9.1844660000	2.5705590000
N	-1.9879480000	9.7386590000	3.5710350000
O	6.5687200000	-1.5081150000	-2.4804870000
C	7.2194930000	-1.7301360000	-1.3403460000
N	6.6207420000	-1.6727130000	-0.1624710000
C	7.3486220000	-1.9119460000	0.9165210000
O	6.8216770000	-1.8943830000	2.1407260000
C	8.5853040000	-2.0271240000	-1.4994510000
C	9.3326740000	-2.2706350000	-0.3639050000
C	8.7203640000	-2.2168760000	0.8735240000
C	9.1674050000	-2.0698570000	-2.8034160000
N	9.6550960000	-2.1074170000	-3.8380870000
H	10.3907820000	-2.5022430000	-0.4427940000
C	9.4436310000	-2.4615160000	2.0808230000
N	10.0412700000	-2.6646240000	3.0356990000
N	0.6129580000	-1.3615970000	3.2637130000
C	-0.6553740000	-0.9682420000	3.2267250000
N	-1.0341240000	0.3057730000	3.2074010000
C	-0.0574180000	1.2067240000	3.2207170000
N	1.2341080000	0.8955820000	3.2553800000
C	1.5271190000	-0.3989550000	3.2554370000
C	-1.7018050000	-2.0175740000	3.1114560000
C	-3.0301350000	-1.6563840000	2.9234740000
C	-3.9829920000	-2.6192700000	2.6367610000
C	-3.5878200000	-3.9399910000	2.5513750000
C	-2.2793160000	-4.3298230000	2.7745060000
C	-1.3363870000	-3.3588510000	3.0539830000
H	-3.2992200000	-0.6052580000	2.9664660000
H	-5.0273610000	-2.3595160000	2.4899450000
H	-2.0199530000	-5.3835770000	2.7365830000
H	-0.2942730000	-3.6243210000	3.2007550000
C	-0.4147940000	2.6448650000	3.1020630000
C	0.5923910000	3.6037080000	3.0793600000
C	0.2971690000	4.9212150000	2.7880400000
C	-1.0146000000	5.2647050000	2.5151620000
C	-2.0397150000	4.3388270000	2.5676060000
C	-1.7320630000	3.0232150000	2.8707350000
H	1.6159890000	3.2924430000	3.2633720000

H	1.0635550000	5.6900860000	2.7745170000
H	-3.0626420000	4.6524970000	2.3783830000
H	-2.5077140000	2.2636490000	2.8876560000
C	2.9555620000	-0.7927640000	3.1312210000
C	3.2972050000	-2.1358570000	3.0312640000
C	4.5831770000	-2.5061890000	2.6792230000
C	5.5131260000	-1.5156370000	2.4263360000
C	5.2144020000	-0.1735030000	2.5761900000
C	3.9266710000	0.1827200000	2.9338810000
H	2.5273000000	-2.8850710000	3.1880580000
H	4.8739820000	-3.5486190000	2.5920770000
H	5.9861360000	0.5724520000	2.4094300000
H	3.6420920000	1.2271860000	3.0189270000
C	-1.0791730000	0.2285770000	0.0025570000
C	0.2944730000	-0.1490550000	0.0111660000
C	1.2987690000	0.8447230000	0.0182100000
C	2.6339400000	0.4527910000	0.0412110000
C	2.9790170000	-0.8874220000	0.0474490000
C	1.9982050000	-1.8634410000	0.0307050000
C	0.6492140000	-1.5166740000	0.0095010000
C	-0.3983640000	-2.5021990000	-0.0156030000
C	-1.6960090000	-2.1469670000	-0.0268150000
C	-2.0835760000	-0.7620680000	-0.0104560000
C	-3.4210250000	-0.3726890000	-0.0107800000
C	-3.7679410000	0.9688180000	0.0039990000
C	-2.7823460000	1.9446730000	0.0125850000
C	-1.4350800000	1.5945520000	0.0082980000
C	-0.3924070000	2.5861960000	-0.0001280000
C	0.9044220000	2.2284950000	0.0056190000
H	3.4124940000	1.2140220000	0.0545600000
H	4.0291400000	-1.1728100000	0.0685090000
H	2.2760760000	-2.9167050000	0.0334920000
H	-0.1151580000	-3.5539340000	-0.0236830000
H	-2.4851280000	-2.9016460000	-0.0461280000
H	-4.1877480000	-1.1465610000	-0.0210370000
H	-4.8163040000	1.2577070000	0.0072430000
H	-3.0445110000	3.0020930000	0.0173420000
H	-0.6869950000	3.6375330000	-0.0024770000
H	1.6870090000	2.9858910000	0.0095090000

Complex cryptand **2** –1,5-dihydroxynaphthalene

131

N	-0.6153290000	-1.2711050000	-3.1684510000
C	-1.3614060000	-0.1746340000	-3.1772240000
N	-0.8712990000	1.0603340000	-3.2246920000
C	0.4518840000	1.1613100000	-3.2188070000
N	1.2725470000	0.1144390000	-3.2123410000
C	0.7004010000	-1.0807950000	-3.1752390000
C	-2.8273660000	-0.3387120000	-3.0047900000
C	-3.6189580000	0.7725020000	-2.7340040000
C	-4.9485540000	0.6169390000	-2.3839270000
C	-5.4738500000	-0.6640230000	-2.3277420000
C	-4.7176750000	-1.7794700000	-2.6354140000
C	-3.3860710000	-1.6104180000	-2.9688560000
H	-3.1625910000	1.7576140000	-2.7672180000
H	-5.5765580000	1.4740750000	-2.1588720000
H	-5.1810330000	-2.7608280000	-2.6042170000
H	-2.7534000000	-2.4676460000	-3.1749310000
C	1.0497000000	2.5125480000	-3.0776040000

C	2.3954260000	2.6294760000	-2.7573110000
C	2.9270740000	3.8502370000	-2.3833160000
C	2.0931140000	4.9506490000	-2.3440290000
C	0.7613520000	4.8730590000	-2.7180700000
C	0.2409910000	3.6452930000	-3.0802890000
H	3.0106110000	1.7357260000	-2.7503080000
H	3.9700070000	3.9432200000	-2.0976530000
H	0.1538060000	5.7728260000	-2.7069540000
H	-0.8114940000	3.5388780000	-3.3255770000
C	1.5825150000	-2.2645480000	-3.0137000000
C	1.0315620000	-3.5065090000	-2.7158010000
C	1.8448430000	-4.5719130000	-2.3766120000
C	3.2152010000	-4.3755240000	-2.3427730000
C	3.7869050000	-3.1601440000	-2.6685760000
C	2.9616160000	-2.1015590000	-3.0024670000
H	-0.0488780000	-3.6134720000	-2.7197750000
H	1.4264840000	-5.5427280000	-2.1300970000
H	4.8681770000	-3.0632770000	-2.6568290000
H	3.3756510000	-1.1233450000	-3.2265860000
O	-6.8231930000	-0.8728600000	-2.0705770000
C	-7.4511030000	-0.3775250000	-1.0038130000
N	-6.7746620000	0.0799190000	0.0321390000
C	-7.4466900000	0.5375530000	1.0717760000
O	-6.8099510000	1.0297340000	2.1363330000
C	-8.8546690000	-0.4090300000	-1.0534690000
C	-9.5487460000	0.0790560000	0.0385620000
C	-8.8495290000	0.5684440000	1.1274950000
C	-9.5256920000	-0.9248070000	-2.2052190000
N	-10.0772670000	-1.3296970000	-3.1224630000
H	-10.6347770000	0.0765980000	0.0419630000
C	-9.5175180000	1.0793730000	2.2830180000
N	-10.0708060000	1.4871610000	3.1978860000
O	2.5826770000	6.2130430000	-2.0274860000
C	3.3782660000	6.4548770000	-0.9830770000
N	3.4372910000	5.6221220000	0.0389220000
C	4.2223020000	5.9204830000	1.0560860000
O	4.3341590000	5.1075820000	2.1104640000
C	4.1060160000	7.6556680000	-1.0357420000
C	4.9376260000	7.9532350000	0.0288550000
C	5.0148950000	7.0801800000	1.0988600000
C	3.9933940000	8.5247090000	-2.1651090000
N	3.9225760000	9.2329930000	-3.0612610000
H	5.5266610000	8.8656640000	0.0240690000
C	5.8593950000	7.3410020000	2.2223450000
N	6.5371480000	7.5697110000	3.1157380000
O	4.0869310000	-5.4278120000	-2.0911950000
C	3.9663670000	-6.2425740000	-1.0417650000
N	3.2339980000	-5.9028590000	0.0009830000
C	3.1572490000	-6.7388260000	1.0180370000
O	2.4167880000	-6.4456180000	2.0876440000
C	4.6855920000	-7.4465330000	-1.1165110000
C	4.5921410000	-8.3196550000	-0.0473250000
C	3.8140220000	-7.9806530000	1.0444310000
C	5.4771720000	-7.7493980000	-2.2676590000
N	6.1161290000	-8.0130660000	-3.1799310000
H	5.1257040000	-9.2653020000	-0.0657890000
C	3.6841780000	-8.8467490000	2.1739450000
N	3.5969750000	-9.5521000000	3.0709490000
N	-0.9208810000	-1.1377050000	3.0972490000
C	-1.3535420000	0.1169090000	3.1213850000

N	-0.5540900000	1.1767920000	3.1706070000
C	0.7499850000	0.9234470000	3.1726240000
N	1.2662580000	-0.3010010000	3.1761060000
C	0.3976280000	-1.3018610000	3.1164360000
C	-2.8155140000	0.3489200000	2.9833840000
C	-3.3165610000	1.6441990000	2.9309920000
C	-4.6497230000	1.8663790000	2.6317090000
C	-5.4649150000	0.7780580000	2.3822340000
C	-5.0008370000	-0.5238760000	2.4729510000
C	-3.6686720000	-0.7316560000	2.7766920000
H	-2.6355230000	2.4757970000	3.0905190000
H	-5.0661870000	2.8680730000	2.5803680000
H	-5.6766750000	-1.3568330000	2.2992930000
H	-3.2608180000	-1.7365420000	2.8222450000
C	1.6895840000	2.0656020000	3.0499670000
C	3.0630200000	1.8637440000	3.1281370000
C	3.9368380000	2.8911780000	2.8187910000
C	3.4188570000	4.1061970000	2.4062160000
C	2.0578620000	4.3504090000	2.3852580000
C	1.1966830000	3.3225600000	2.7178310000
H	3.4347120000	0.8808130000	3.4031470000
H	5.0141220000	2.7655580000	2.8746320000
H	1.6809710000	5.3246310000	2.0911110000
H	0.1217020000	3.4643580000	2.6735340000
C	0.9357700000	-2.6776610000	2.9542960000
C	0.0695870000	-3.7340310000	2.7034360000
C	0.5684550000	-4.9866340000	2.3880130000
C	1.9370380000	-5.1601850000	2.3263860000
C	2.8214080000	-4.1302810000	2.5967960000
C	2.3114310000	-2.8846610000	2.9109710000
H	-1.0006450000	-3.5536400000	2.7328850000
H	-0.0860700000	-5.8304100000	2.1916140000
H	3.8920210000	-4.3099200000	2.5531130000
H	2.9720680000	-2.0424560000	3.0937960000
C	0.2531130000	-1.4097960000	-0.0129340000
C	-1.5777730000	0.2557520000	-0.0363500000
O	-2.4419910000	-0.7927380000	-0.0147050000
C	-0.1925140000	-0.0668920000	-0.0308980000
C	-1.9923210000	1.5587490000	-0.0540270000
C	0.7368820000	0.9964800000	-0.0370650000
C	1.5892750000	-1.6780490000	0.0200780000
C	2.1214450000	0.6688680000	0.0084310000
C	-1.0470670000	2.6032660000	-0.0806930000
C	2.5355280000	-0.6343070000	0.0405890000
C	0.2919100000	2.3379400000	-0.0736490000
H	1.9385780000	-2.7110800000	0.0258370000
O	2.9858870000	1.7188980000	0.0266890000
H	3.5995250000	-0.8653820000	0.0698670000
H	-3.0598450000	1.7757440000	-0.0406260000
H	-1.3951180000	3.6335970000	-0.1176520000
H	-0.4843310000	-2.2078000000	-0.0212720000
H	-3.3568210000	-0.4823390000	-0.0484690000
H	1.0299100000	3.1372630000	-0.1112840000
H	3.8720890000	1.4210140000	0.2477680000

In DMSO solvent:

Cryptand **2**:

111

N	1.13661	0.08508	1.77178
C	0.27408	1.09578	1.78610
N	-1.04503	0.93656	1.82472
C	-1.48348	-0.31576	1.90195
N	-0.68500	-1.37750	1.92491
C	0.61911	-1.13621	1.85360
C	0.82138	2.47700	1.83334
C	-0.01475	3.54755	2.13565
C	0.50743	4.82367	2.28810
C	1.86721	5.00131	2.13468
C	2.72093	3.95993	1.81443
C	2.18888	2.69210	1.66538
H	-1.08270	3.37664	2.26941
H	-0.13097	5.67886	2.53288
H	3.79185	4.15145	1.68519
H	2.84047	1.85189	1.42521
C	-2.94647	-0.54325	2.00317
C	-3.46656	-1.82041	1.80172
C	-4.82982	-2.03753	1.88167
C	-5.65081	-0.96284	2.16560
C	-5.16524	0.30855	2.39375
C	-3.79763	0.51511	2.30636
H	-2.79411	-2.64556	1.56780
H	-5.26710	-3.02475	1.69721
H	-5.85793	1.12784	2.61346
H	-3.38341	1.50980	2.46993
C	1.55350	-2.28902	1.93127
C	2.92581	-2.08471	1.79270
C	3.80494	-3.14484	1.91608
C	3.29067	-4.40295	2.17181
C	1.93829	-4.63848	2.31104
C	1.06656	-3.56651	2.19156
H	3.30444	-1.08329	1.58906
H	4.88600	-3.01076	1.79963
H	1.57685	-5.65280	2.50984
H	-0.00577	-3.72143	2.30697
O	2.39237	6.28769	2.33792
C	2.76938	6.98102	1.26231
N	2.61513	6.43990	0.08000
C	2.94942	7.10291	-0.99997
O	2.78523	6.51692	-2.18897
C	3.30926	8.26611	1.43675
C	3.67239	8.97051	0.29937
C	3.49276	8.39614	-0.94974
C	3.46811	8.80648	2.74697
N	3.59312	9.23394	3.80241
H	4.09904	9.97376	0.38654
C	3.84877	9.07364	-2.15283
N	4.13266	9.61240	-3.12262
O	-7.04111	-1.16346	2.18172
C	-7.64267	-1.15250	0.99024
N	-6.90875	-0.95647	-0.07924
C	-7.46508	-0.94266	-1.26544
O	-6.69228	-0.75412	-2.33482
C	-9.03094	-1.34951	0.92629
C	-9.62615	-1.33188	-0.32563
C	-8.84522	-1.12537	-1.45202
C	-9.78100	-1.55885	2.12058
N	-10.37614	-1.72660	3.08399
H	-10.70519	-1.48162	-0.42414

C	-9.40058	-1.10432	-2.76519
N	-9.84033	-1.08648	-3.82253
O	4.17319	-5.48846	2.29876
C	4.66377	-6.00475	1.17052
N	4.31984	-5.46013	0.02886
C	4.78249	-5.94316	-1.09811
O	4.42015	-5.35856	-2.24126
C	5.52485	-7.11148	1.24768
C	6.01614	-7.62882	0.05924
C	5.64832	-7.04820	-1.14485
C	5.87014	-7.66601	2.51514
N	6.14361	-8.10687	3.53516
H	6.68882	-8.49104	0.07131
C	6.11985	-7.53861	-2.39785
N	6.48930	-7.92099	-3.39038
N	1.50270	0.29659	-1.91414
C	0.64993	1.31488	-1.93897
N	-0.67078	1.17119	-1.90230
C	-1.12506	-0.07541	-1.82535
N	-0.33878	-1.14563	-1.77957
C	0.96912	-0.91904	-1.85633
C	1.19938	2.68865	-2.04799
C	0.38348	3.78617	-1.77737
C	0.89360	5.06776	-1.85217
C	2.22719	5.22890	-2.18584
C	3.04858	4.16587	-2.49818
C	2.52317	2.88398	-2.42801
H	-0.65643	3.62710	-1.49196
H	0.28019	5.94353	-1.61490
H	4.09366	4.34424	-2.77149
H	3.15126	2.02359	-2.65809
C	-2.59584	-0.27928	-1.86228
C	-3.11469	-1.49636	-2.29292
C	-4.48451	-1.66359	-2.43404
C	-5.31074	-0.60009	-2.13286
C	-4.82505	0.61321	-1.67805
C	-3.45694	0.77155	-1.54949
H	-2.43683	-2.31377	-2.53914
H	-4.91393	-2.60626	-2.78888
H	-5.52186	1.42193	-1.43203
H	-3.04596	1.72193	-1.20642
C	1.88090	-2.08962	-1.92707
C	3.23911	-1.90107	-2.16620
C	4.09001	-2.99036	-2.27599
C	3.55600	-4.25565	-2.14389
C	2.20946	-4.47563	-1.91660
C	1.37073	-3.38158	-1.80617
H	3.63303	-0.89064	-2.27027
H	5.16303	-2.86501	-2.45461
H	1.83447	-5.49939	-1.81074
H	0.30653	-3.52596	-1.62173

Complex cryptand **2** – anthracene:

135

N	-1.01802	1.02207	3.22570
C	-1.57331	-0.18327	3.17690
N	-0.87389	-1.31435	3.14192
C	0.44779	-1.19040	3.12485
N	1.07852	-0.02032	3.16031

C	0.31060	1.06256	3.19210
C	-3.05064	-0.26524	3.03496
C	-3.65956	-1.49093	2.77195
C	-5.00045	-1.55272	2.43474
C	-5.71503	-0.36870	2.36722
C	-5.14805	0.85654	2.66118
C	-3.80582	0.90217	2.99588
H	-3.06338	-2.40257	2.79534
H	-5.49374	-2.50601	2.21651
H	-5.76045	1.76283	2.61633
H	-3.32639	1.86030	3.19315
C	1.25990	-2.42266	2.95074
C	2.59602	-2.32041	2.57385
C	3.33699	-3.45125	2.27874
C	2.71966	-4.68531	2.37275
C	1.40213	-4.82099	2.77598
C	0.67068	-3.67989	3.05757
H	3.05287	-1.33884	2.45658
H	4.38148	-3.37420	1.95838
H	0.96021	-5.81944	2.85302
H	-0.37563	-3.76279	3.35073
C	0.97500	2.38601	3.06906
C	0.22566	3.51496	2.74254
C	0.85356	4.70752	2.42376
C	2.23763	4.74797	2.44975
C	3.00319	3.65757	2.81605
C	2.36278	2.46944	3.11881
H	-0.86141	3.44863	2.70321
H	0.28069	5.60044	2.15133
H	4.09420	3.74472	2.84184
H	2.94752	1.58316	3.36425
O	-7.08536	-0.38139	2.11038
C	-7.61452	-0.87187	0.99178
N	-6.86365	-1.14739	-0.05705
C	-7.45284	-1.61753	-1.13936
O	-6.74491	-1.95012	-2.21633
C	-9.01060	-1.04043	1.00451
C	-9.62379	-1.53070	-0.13284
C	-8.83943	-1.83236	-1.23006
C	-9.75338	-0.71284	2.17777
N	-10.35306	-0.44976	3.12172
H	-10.70703	-1.67790	-0.16311
C	-9.40019	-2.34333	-2.43799
N	-9.84844	-2.75109	-3.39992
O	3.42201	-5.86489	2.14297
C	4.22478	-6.04666	1.09321
N	4.11353	-5.29458	0.01750
C	4.87162	-5.56356	-1.02496
O	4.79745	-4.83854	-2.14104
C	5.13474	-7.11241	1.19095
C	5.94610	-7.38198	0.10377
C	5.82126	-6.60138	-1.03026
C	5.19981	-7.88436	2.38918
N	5.25249	-8.50533	3.35306
H	6.66972	-8.20130	0.13890
C	6.61480	-6.83436	-2.19225
N	7.24179	-7.02072	-3.11039
O	2.92395	5.93959	2.21999
C	2.79417	6.66528	1.11257
N	2.22139	6.17134	0.03143

C	2.13901	6.94123	-1.03671
O	1.53267	6.51927	-2.14312
C	3.33337	7.96272	1.17340
C	3.24944	8.76362	0.05018
C	2.63978	8.25485	-1.08070
C	3.94082	8.42242	2.37903
N	4.42221	8.79050	3.33265
H	3.65518	9.77916	0.05708
C	2.51609	9.02085	-2.27733
N	2.41889	9.63220	-3.23125
N	-1.10250	0.84318	-3.16676
C	-1.40168	-0.44847	-3.13028
N	-0.48666	-1.41310	-3.11708
C	0.78278	-1.02573	-3.12183
N	1.16578	0.24824	-3.14959
C	0.19201	1.15233	-3.14535
C	-2.82988	-0.84116	-2.98768
C	-3.15096	-2.17070	-2.73400
C	-4.45400	-2.53458	-2.43931
C	-5.42087	-1.54875	-2.40371
C	-5.13652	-0.21973	-2.66547
C	-3.82895	0.12898	-2.95841
H	-2.36455	-2.92453	-2.74149
H	-4.72910	-3.57478	-2.23733
H	-5.93830	0.52678	-2.63941
H	-3.57184	1.17249	-3.14011
C	1.82829	-2.07220	-2.98265
C	3.16371	-1.78920	-3.24381
C	4.14414	-2.72782	-2.96420
C	3.77057	-3.93367	-2.40087
C	2.44363	-4.26269	-2.17561
C	1.47584	-3.32213	-2.47545
H	3.44160	-0.81286	-3.64220
H	5.20339	-2.52114	-3.14755
H	2.17422	-5.24007	-1.76553
H	0.42645	-3.54598	-2.28409
C	0.55965	2.58560	-2.99684
C	-0.43892	3.53007	-2.78107
C	-0.11068	4.83801	-2.46594
C	1.22463	5.17676	-2.37003
C	2.24211	4.26831	-2.60773
C	1.89872	2.96578	-2.92371
H	-1.48443	3.23102	-2.84677
H	-0.87913	5.59828	-2.29334
H	3.28863	4.58602	-2.53813
H	2.67843	2.22091	-3.08201
C	1.04644	-0.86906	-0.01577
C	-0.13337	-0.13242	0.01413
C	-1.41924	-0.76095	0.02102
C	-2.55195	-0.01843	0.00700
C	-2.48850	1.40692	0.01202
C	-1.28933	2.04105	0.03273
C	-0.06978	1.29424	0.01900
C	1.16956	1.92150	-0.00645
C	2.35023	1.18624	-0.05216
C	3.62722	1.82675	-0.09997
C	4.76762	1.09240	-0.15087
C	4.70698	-0.33420	-0.16727
C	3.51129	-0.97477	-0.12417
C	2.28852	-0.23821	-0.05771

H	0.99472	-1.96201	-0.00393
H	-1.46335	-1.85417	0.02134
H	-3.53029	-0.50671	-0.00268
H	-3.41752	1.97998	0.01157
H	-1.22645	3.13142	0.04186
H	1.21847	3.01642	-0.00116
H	3.65590	2.91973	-0.09256
H	5.73943	1.58679	-0.18801
H	5.63332	-0.90738	-0.23217
H	3.45709	-2.06573	-0.16359

Complex cryptand **2** – pyrene:

137

N	-1.06760	-0.80493	3.27821
C	0.20148	-1.20084	3.25086
N	1.23148	-0.36056	3.23345
C	0.93709	0.93679	3.22735
N	-0.30503	1.40724	3.24635
C	-1.28190	0.50545	3.24935
C	0.47413	-2.65920	3.13038
C	1.77373	-3.11242	2.92334
C	2.01428	-4.43998	2.60703
C	0.93622	-5.30018	2.51605
C	-0.36145	-4.89102	2.76675
C	-0.58643	-3.56052	3.07199
H	2.60238	-2.40635	2.96712
H	3.02913	-4.81292	2.43021
H	-1.18023	-5.61579	2.71623
H	-1.60337	-3.20389	3.23213
C	2.05415	1.91250	3.10128
C	1.78107	3.27506	2.98733
C	2.78998	4.17055	2.68258
C	4.06961	3.67827	2.49053
C	4.37959	2.34118	2.63799
C	3.35890	1.45689	2.94859
H	0.75695	3.62790	3.10372
H	2.59693	5.24409	2.58656
H	5.41240	2.00208	2.50588
H	3.57175	0.39182	3.03279
C	-2.68456	0.98737	3.12092
C	-3.73434	0.07479	3.05619
C	-5.01708	0.49864	2.75069
C	-5.22568	1.84407	2.50683
C	-4.21082	2.77842	2.59510
C	-2.93501	2.33995	2.90770
H	-3.53542	-0.98474	3.21445
H	-5.85635	-0.20289	2.69823
H	-4.42768	3.83742	2.42103
H	-2.11085	3.05155	2.94834
O	1.16011	-6.66287	2.31351
C	1.42638	-7.23125	1.14172
N	1.39910	-6.55309	0.00695
C	1.65326	-7.21072	-1.11166
O	1.64868	-6.61355	-2.29963
C	1.71610	-8.60710	1.21205
C	1.98490	-9.28625	0.04042
C	1.95622	-8.58502	-1.14857
C	1.72212	-9.26703	2.47665
N	1.72744	-9.80156	3.48989

H	2.21416	-10.35531	0.05382
C	2.22232	-9.21841	-2.39842
N	2.43750	-9.73066	-3.40016
O	5.11409	4.58030	2.27892
C	5.46102	5.06019	1.08897
N	4.85701	4.67868	-0.02320
C	5.25448	5.22499	-1.15949
O	4.68934	4.91951	-2.32325
C	6.50944	5.99922	1.11648
C	6.91952	6.56510	-0.07423
C	6.28491	6.18045	-1.23873
C	7.11667	6.34639	2.35960
N	7.60820	6.62591	3.35592
H	7.72758	7.30159	-0.09463
C	6.65126	6.72373	-2.50564
N	6.94799	7.16327	-3.52106
O	-6.53128	2.30021	2.30979
C	-7.15342	2.34040	1.13549
N	-6.55070	1.99982	0.00982
C	-7.24865	2.06842	-1.11059
O	-6.73067	1.72591	-2.28614
C	-8.49146	2.77394	1.19457
C	-9.21435	2.84409	0.02025
C	-8.59154	2.48726	-1.15937
C	-9.06751	3.12493	2.45134
N	-9.53334	3.40804	3.45890
H	-10.25651	3.17545	0.02423
C	-9.27359	2.53605	-2.41095
N	-9.82450	2.57647	-3.41444
N	-1.05466	-0.94628	-3.26092
C	0.23863	-1.25002	-3.23279
N	1.20617	-0.33751	-3.21438
C	0.82028	0.93470	-3.20778
N	-0.45320	1.31523	-3.23365
C	-1.36256	0.34666	-3.23901
C	0.61843	-2.68573	-3.12420
C	1.95156	-3.03850	-2.94595
C	2.29696	-4.34470	-2.63822
C	1.28992	-5.28150	-2.51784
C	-0.04116	-4.97413	-2.74220
C	-0.37030	-3.66545	-3.04628
H	2.72381	-2.27241	-3.00252
H	3.33957	-4.64074	-2.48246
H	-0.80326	-5.75708	-2.67044
H	-1.41406	-3.38566	-3.18543
C	1.86257	1.99085	-3.08872
C	1.48875	3.33102	-3.01988
C	2.42549	4.30661	-2.72926
C	3.73458	3.91776	-2.50654
C	4.14431	2.60168	-2.60427
C	3.19564	1.63680	-2.90301
H	0.44414	3.60481	-3.16335
H	2.15473	5.36568	-2.67237
H	5.19681	2.34259	-2.44623
H	3.48458	0.58775	-2.95555
C	-2.79703	0.72526	-3.11833
C	-3.76936	-0.26496	-3.01641
C	-5.07743	0.06468	-2.70360
C	-5.39118	1.39501	-2.49683
C	-4.45701	2.40634	-2.63035

C	-3.15322	2.06044	-2.94394
H	-3.48760	-1.30950	-3.14534
H	-5.85757	-0.69832	-2.61668
H	-4.75586	3.45031	-2.48669
H	-2.38850	2.83305	-3.01766
C	1.17464	-0.41879	0.00194
C	-0.19736	-0.03340	0.00156
C	-0.54441	1.33685	0.00010
C	-1.89052	1.69285	-0.01028
C	-2.87608	0.72003	-0.00366
C	-2.54456	-0.62342	0.00539
C	-1.20792	-1.02093	0.00084
C	-0.82143	-2.40677	-0.01506
C	0.47385	-2.76948	-0.01578
C	1.52327	-1.78627	0.00057
C	2.87047	-2.14524	0.01020
C	3.86136	-1.17445	0.00761
C	3.52158	0.17156	-0.00013
C	2.18552	0.56727	0.00292
C	1.80218	1.95436	0.01250
C	0.50745	2.31944	0.01020
H	-2.16249	2.75086	-0.02020
H	-3.92477	1.01801	-0.01403
H	-3.32724	-1.38566	0.00537
H	-1.61076	-3.16225	-0.03047
H	0.76002	-3.82324	-0.03435
H	3.13037	-3.20691	0.01363
H	4.91165	-1.46970	0.01060
H	4.29711	0.94144	-0.00330
H	2.59737	2.70557	0.02134
H	0.22622	3.37557	0.01549

Complex cryptand **2** –1,5-dihydroxynaphthalene

131

N	-0.61533	1.27110	3.16845
C	-1.36141	0.17464	3.17722
N	-0.87130	-1.06033	3.22469
C	0.45188	-1.16131	3.21881
N	1.27255	-0.11444	3.21234
C	0.70040	1.08079	3.17524
C	-2.82737	0.33871	3.00479
C	-3.61896	-0.77250	2.73400
C	-4.94855	-0.61694	2.38393
C	-5.47385	0.66403	2.32774
C	-4.71767	1.77947	2.63541
C	-3.38607	1.61042	2.96886
H	-3.16259	-1.75761	2.76722
H	-5.57656	-1.47407	2.15887
H	-5.18103	2.76083	2.60422
H	-2.75340	2.46765	3.17493
C	1.04970	-2.51255	3.07760
C	2.39542	-2.62948	2.75731
C	2.92707	-3.85024	2.38332
C	2.09311	-4.95065	2.34403
C	0.76135	-4.87306	2.71807
C	0.24099	-3.64529	3.08029
H	3.01061	-1.73573	2.75031
H	3.97000	-3.94322	2.09765
H	0.15380	-5.77283	2.70695

H	-0.81150	-3.53888	3.32558
C	1.58252	2.26455	3.01370
C	1.03157	3.50651	2.71580
C	1.84485	4.57191	2.37661
C	3.21520	4.37552	2.34277
C	3.78691	3.16014	2.66858
C	2.96162	2.10156	3.00247
H	-0.04888	3.61347	2.71977
H	1.42649	5.54273	2.13010
H	4.86818	3.06327	2.65683
H	3.37565	1.12334	3.22659
O	-6.82319	0.87287	2.07058
C	-7.45110	0.37753	1.00381
N	-6.77466	-0.07991	-0.03214
C	-7.44669	-0.53755	-1.07178
O	-6.80995	-1.02973	-2.13633
C	-8.85467	0.40904	1.05347
C	-9.54875	-0.07905	-0.03856
C	-8.84953	-0.56844	-1.12749
C	-9.52569	0.92481	2.20522
N	-10.07727	1.32970	3.12246
H	-10.63478	-0.07659	-0.04196
C	-9.51752	-1.07937	-2.28302
N	-10.07081	-1.48715	-3.19789
O	2.58267	-6.21305	2.02749
C	3.37826	-6.45488	0.98308
N	3.43729	-5.62212	-0.03892
C	4.22230	-5.92049	-1.05609
O	4.33416	-5.10759	-2.11046
C	4.10601	-7.65567	1.03574
C	4.93762	-7.95324	-0.02885
C	5.01489	-7.08018	-1.09886
C	3.99339	-8.52471	2.16511
N	3.92257	-9.23300	3.06126
H	5.52665	-8.86567	-0.02407
C	5.85939	-7.34101	-2.22234
N	6.53714	-7.56972	-3.11574
O	4.08694	5.42781	2.09119
C	3.96637	6.24257	1.04177
N	3.23400	5.90286	-0.00098
C	3.15725	6.73882	-1.01804
O	2.41679	6.44562	-2.08764
C	4.68560	7.44653	1.11651
C	4.59215	8.31965	0.04732
C	3.81403	7.98065	-1.04443
C	5.47718	7.74939	2.26766
N	6.11613	8.01306	3.17993
H	5.12571	9.26530	0.06579
C	3.68418	8.84675	-2.17394
N	3.59698	9.55210	-3.07095
N	-0.92088	1.13771	-3.09725
C	-1.35354	-0.11691	-3.12139
N	-0.55409	-1.17679	-3.17061
C	0.74998	-0.92345	-3.17262
N	1.26626	0.30100	-3.17611
C	0.39763	1.30186	-3.11644
C	-2.81551	-0.34892	-2.98338
C	-3.31656	-1.64420	-2.93099
C	-4.64972	-1.86637	-2.63171
C	-5.46492	-0.77805	-2.38223

C	-5.00084	0.52388	-2.47295
C	-3.66867	0.73166	-2.77669
H	-2.63552	-2.47580	-3.09052
H	-5.06619	-2.86807	-2.58037
H	-5.67667	1.35684	-2.29929
H	-3.26082	1.73654	-2.82225
C	1.68958	-2.06560	-3.04997
C	3.06302	-1.86375	-3.12814
C	3.93684	-2.89118	-2.81879
C	3.41885	-4.10620	-2.40622
C	2.05786	-4.35041	-2.38526
C	1.19668	-3.32256	-2.71783
H	3.43471	-0.88082	-3.40315
H	5.01412	-2.76556	-2.87463
H	1.68097	-5.32463	-2.09111
H	0.12170	-3.46436	-2.67353
C	0.93577	2.67766	-2.95430
C	0.06959	3.73403	-2.70344
C	0.56846	4.98663	-2.38801
C	1.93704	5.16018	-2.32639
C	2.82141	4.13028	-2.59680
C	2.31143	2.88466	-2.91097
H	-1.00064	3.55364	-2.73289
H	-0.08607	5.83041	-2.19161
H	3.89202	4.30992	-2.55311
H	2.97207	2.04245	-3.09380
C	0.25311	1.40980	0.01293
C	-1.57777	-0.25575	0.03635
O	-2.44199	0.79274	0.01470
C	-0.19251	0.06689	0.03090
C	-1.99232	-1.55875	0.05403
C	0.73688	-0.99648	0.03707
C	1.58928	1.67805	-0.02008
C	2.12144	-0.66887	-0.00843
C	-1.04707	-2.60326	0.08069
C	2.53553	0.63431	-0.04059
C	0.29191	-2.33794	0.07365
H	1.93858	2.71108	-0.02584
O	2.98589	-1.71890	-0.02669
H	3.59953	0.86538	-0.06987
H	-3.05985	-1.77574	0.04063
H	-1.39512	-3.63360	0.11765
H	-0.48433	2.20780	0.02127
H	-3.35682	0.48234	0.04847
H	1.02991	-3.13726	0.11128
H	3.87209	-1.42102	-0.24777
